# Roles of gut microbiome in epilepsy risk: A Mendelian randomization study

**DOI:** 10.3389/fmicb.2023.1115014

**Published:** 2023-02-27

**Authors:** Youjie Zeng, Si Cao, Heng Yang

**Affiliations:** ^1^Department of Anesthesiology, Third Xiangya Hospital, Central South University, Changsha, Hunan, China; ^2^Department of Neurology, Third Xiangya Hospital, Central South University, Changsha, Hunan, China

**Keywords:** gut microbiome, epilepsy, causal relationship, incidence risk, Mendelian randomization, MiBioGen, FinnGen, microbiota-gut-brain axis

## Abstract

**Background:**

Recent studies have suggested an association between gut microbiomes (GMs) and epilepsy. However, the GM taxa identified in different studies are variable. In addition, observational studies cannot indicate causality. Therefore, our study aimed to explore the causal association of GMs with epilepsy and identify the most influential GM taxa.

**Methods:**

We conducted a Mendelian randomization (MR) study using summary statistics from genome-wide association studies (GWAS) of 211 GM taxa and epilepsy. The GWAS summary statistics for 211 GM taxa (from phylum to genus level) were generated by the MiBioGen consortium, while the FinnGen consortium provided the GWAS summary statistics for epilepsy. The primary analytical method to assess causality was the inverse-variance weighted (IVW) approach. To complement the IVW method, we also applied four additional MR methods: MR-Egger, weighted median, simple mode, and weighted. In addition, we conducted sensitivity analyses using Cochrane’s *Q*-test, MR-Egger intercept test, MR-PRESSO global test, and leave-one-out analysis.

**Results:**

We evaluated the causal effect of 211 GM taxa (from phylum to genus level) on epilepsy, generalized epilepsy, and focal epilepsy. After using the Bonferroni method for multiple testing correction, *Class Betaproteobacteria* [odds ratio (OR) = 1.357, 95% confidence interval (CI): 1.126–1.635, *p* = 0.001] and *Order Burkholderiales* (OR = 1.336, 95% CI: 1.112–1.606, *p* = 0.002). In addition, 21 nominally significant causal relationships were also identified. Further, the MR-Egger intercept test and MR-PRESSO global test suggested that our MR analysis was unaffected by horizontal pleiotropy (*p* > 0.05). Finally, the leave-one-out analysis suggested the robustness of the results.

**Conclusion:**

Through the MR study, we analyzed the causal relationship of 211 GM taxa with epilepsy and determined the specific intestinal flora associated with increased epilepsy risk. Our findings may provide helpful biomarkers for disease progression and potential candidate therapeutic targets for epilepsy. In addition, in-depth analysis of large-scale microbiome GWAS datasets based on metagenomics sequencing is necessary for future studies.

## 1. Introduction

Epilepsy is a common, chronic neurological disorder characterized by sudden abnormal excessive ultra-synchronized neuron discharges that result in temporary involuntary brain dysfunction (Fisher et al., 2005). Globally, there are 70 million people with epilepsy, with the highest incidence in infants and the elderly, posing a tremendous social burden throughout the world (Collaborators, 2019; Thijs et al., 2019). Despite advances and innovations in antiepileptic medications, approximately one-third of the patients suffer from drug-resistant epilepsy (de Biase et al., 2019). Therefore, further insights into the pathogenesis and the exploration of novel therapeutic targets for epilepsy are required.

There is growing evidence that the gut microbiome (GM) can regulate host homeostasis, including cardiovascular function, metabolism, and immune/inflammatory response (Le Chatelier et al., 2013). Recent research has shown that GMs play a role in neuropsychiatric disorders (Iannone et al., 2019), as they regulate brain function and behavior *via* the microbiota-gut-brain axis (Johnson and Foster, 2018). Differences in GM taxa have been identified in epilepsy patients compared to controls (Dong et al., 2022). The ketogenic diet (KD) is a treatment approach for intractable epilepsy (D’Andrea Meira et al., 2019). During the KD treatment of drug-resistant epilepsy, the GM pattern was altered simultaneously (Lindefeldt et al., 2019). Consequently, GMs may be involved in the crosstalk between KD and epilepsy (Fan et al., 2019). Furthermore, researchers are investigating the possibility of using the change in GM composition as a surrogate marker for the efficacy of the KD treatment in patients with drug-resistant epilepsy (Thambi et al., 2020). However, the effect of various GM taxa on epilepsy has not yet been determined. The 16S rRNA and metagenomic sequencing are the most widely used methods for identifying GM taxonomic characteristics (Durazzi et al., 2021), providing the basis for identifying the potential role of GM taxa. Recent research has increasingly focused on the causal effects of GMs on epilepsy, particularly refractory epilepsy (Lum et al., 2020). In addition, perturbations for certain GM taxa levels have been reported to affect the activity of epileptic neurons (Darch and McCafferty, 2022).

Nevertheless, the specific contribution of various GM taxa to epilepsy warrants further exploration. Similar to randomized controlled trials (RCT), the Mendelian randomization (MR) study is a novel research method for exploring the causal association between exposure and outcome (Swanson et al., 2017). In MR studies, single nucleotide polymorphisms (SNPs) are considered instrumental variables (IVs) to estimate the causal association between exposures and the outcomes of interest (Burgess et al., 2017). SNPs conform to the principle of random assignment of genetic variants at meiosis, which avoids the effect of confounding factors and the potential impact of reverse causation since genetic variants precede the onset of disease (Lawlor et al., 2008). Therefore, the causal associations of exposure factors of interest to outcomes can be identified more rapidly by MR analysis compared to RCT. For example, a recent MR study by Cai et al. has identified several blood metabolites with potential causal associations with epilepsy (Cai et al., 2022). Here, we conducted an MR study using large-scale GWAS summary statistics of GMs and epilepsy to identify potentially influential GM taxa, which could provide confidence to some existing evidence and may yield new insights into the prevention and treatment of epilepsy.

## 2. Materials and methods

### 2.1. Study design

**Figure 1:**
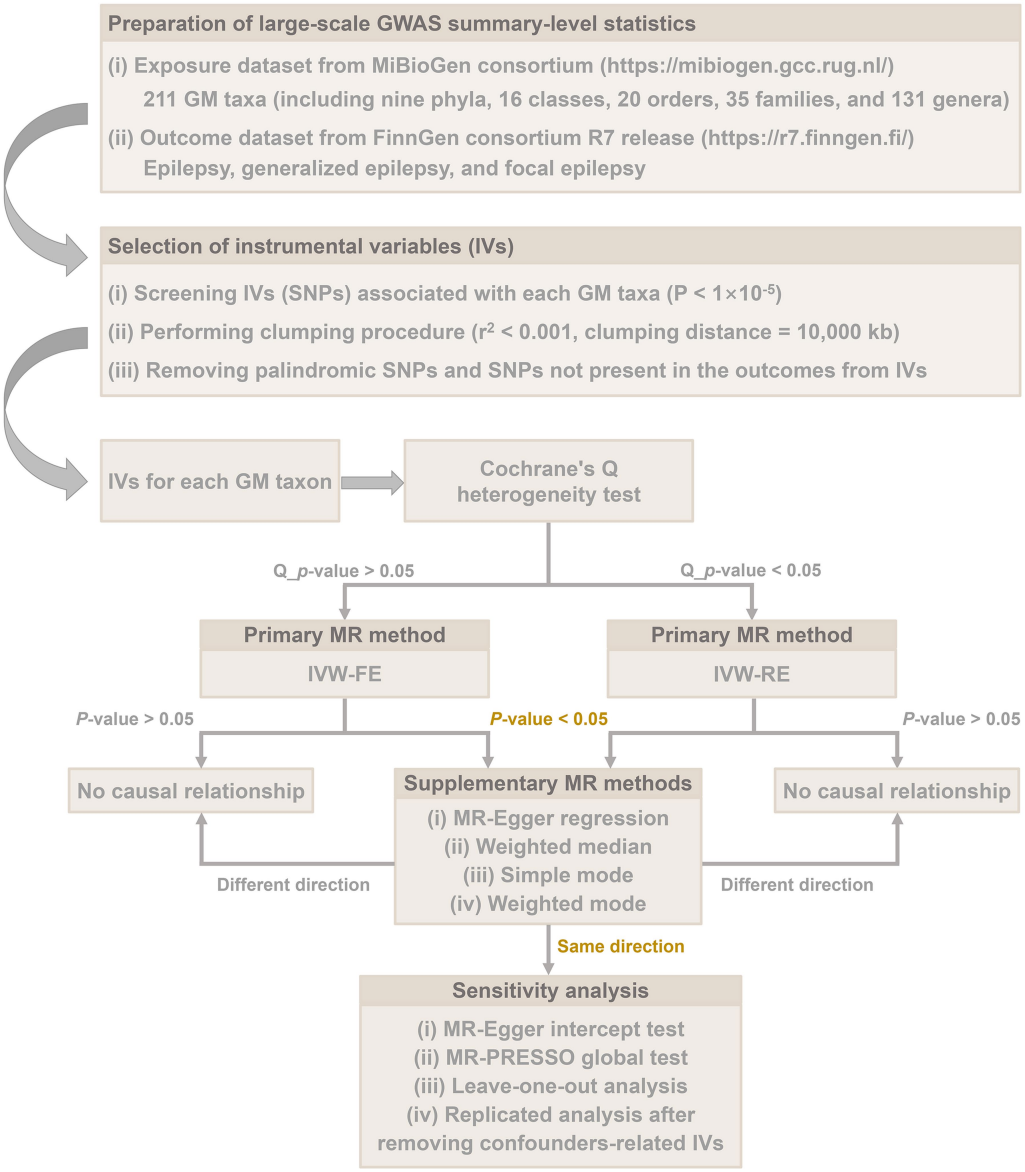
Overall flow chart of this study.

The overall flow chart of this study is shown in Figure 1. MR studies are required to satisfy the following three assumptions: (i) IVs are strongly associated with exposure factors, (ii) IVs are independent of confounding factors, and (iii) IVs are solely associated with outcomes through exposure factors (Burgess et al., 2017). Specifically, we identified GM taxa that have a causal effect on epilepsy, generalized epilepsy, and focal epilepsy by performing a two-sample MR analysis. Our results were reported in accordance with the STROBE-MR guidelines (Skrivankova et al., 2021).

### 2.2. Data sources for the exposure

A study from the MiBioGen consortium analyzed the host genotypes and 16S fecal microbiomes rRNA gene sequencing profiles of 18,340 participants (Kurilshikov et al., 2021). This GWAS study examined 211 GM taxa (from genus to phylum level) and ultimately identified genetic variants associated with nine phyla, 16 classes, 20 orders, 35 families, and 131 genera. The GWAS summary statistics of GMs are available for download at^1^ (Swertz and Jansen, 2007; Swertz et al., 2010; van der Velde et al., 2019).

### 2.3. Data sources for the outcome

We obtained GWAS summary statistics for epilepsy from the FinnGen consortium R7 release^2^ (Kurki et al., 2022). In addition, we downloaded GWAS summary data for generalized epilepsy and focal epilepsy. Epilepsy diagnosis in FinnGen was based on G40 in the 10th version of the International Classification of Diseases (ICD). Cases of generalized and focal epilepsy were narrower endpoints of epilepsy under the strict definition. Table 1 shows the details of the exposure and outcome analyzed in this MR study.

**Table 1:** Details of the exposure and outcome.

Trait	Consortium	Samples	Case	Control
**Exposure**				
211 GM taxa	MiBioGen	18,340	/	/
**Outcome**				
Epilepsy	FinnGen (R7)	252,026	8,523	243,503
Generalized epilepsy	FinnGen (R7)	302,828	2,197	300,631
Focal epilepsy	FinnGen (R7)	301,493	862	300,631

GM, gut microbiome.

### 2.4. Identification of IVs

SNPs strongly associated with each GM taxon were used as IVs in this MR study. Since the number of IVs obtained under the strict threshold (*p* < 5 × 10^−8^) was extremely minimal, we adopted a more comprehensive threshold (*p* < 1 × 10^−5^) to obtain relatively more IVs to achieve relatively robust results. In addition, to ensure each IV’s independence, SNPs within a window size of 10,000 kb at a threshold of *r*^2^ < 0.001 were pruned to mitigate linkage disequilibrium (LD). Then, palindromic SNPs and SNPs not present in the outcome were removed from the IVs. Finally, we calculated the F-statistic of IVs to assess the degree of weak instrumental bias. If the F-statistic >10, it was considered that no bias was caused by weak IVs (Pierce et al., 2011).

### 2.5. Statistical methods

The inverse variance weighted fixed-effect (IVW-FE) method or the IVW random effect (IVW-RE) method was used as the primary MR method for inferring causality. The choice of IVW-FE or IVW-RE was determined based on Cochrane’s Q heterogeneity test. The IVW method is an extension of the Wald ratio estimator based on the principles of Meta-analysis (Pagoni et al., 2019).

For each GM taxon, if the IVW method identified a causal association (*p* < 0.05), four additional MR methods, MR-Egger, weighted median, simple mode, and weighted mode, would be performed to supplement the IVW result (Bowden et al., 2016; Burgess and Thompson, 2017). The criterion for using the weighted median method is that at least 50% of the SNPs must satisfy the premise that they are valid IVs (Bowden et al., 2016). The MR-Egger method provides unbiased estimates even when all selected IVs are multivariate (Burgess and Thompson, 2017). Finally, the results of causal associations were presented as odds ratios (OR) and 95% confidence intervals (95% CI). The significance threshold was set at *p* < 0.05. In addition, the Bonferroni method was used for multiple testing corrections. The threshold for various levels was *p* < 0.05/n, where n represents the number of taxa at a particular level.

Only exposure-outcome pairs with the same direction identified by all MR methods were considered to have a causal association. To test the stability of the causal association, we further performed several sensitivity analyses. First, the MR-Egger intercept test and MR-PRESSO global test were utilized to detect horizontal pleiotropy (Rees et al., 2017; Verbanck et al., 2018). In addition, the leave-one-out analysis was performed to assess the robustness of the results. Furthermore, we performed replicated MR analyses after excluding potential confounders from the IVs. Specifically, the confounders-related SNPs were retrieved from the PhenoScanner V2 database^3^ (Staley et al., 2016; Kamat et al., 2019), including education level (Wang et al., 2021), diabetes (Marcovecchio et al., 2015), obesity (Hafizi et al., 2017), and smoking (Yuan et al., 2021).

All analyses in this study were performed based on R software(version 4.2.1). The “TwoSampleMR” R package^4^ and the “MRPRESSO” R package^5^ were used in our MR study.

## 3. Results

### 3.1. Details of IVs

Overall, 2,252 SNPs were identified as final IVs. These SNPs were classified according to five levels: phylum, class, order, family, and genus. Specifically, there were 102 IVs in 9 phyla, 179 IVs in 16 classes, 216 IVs in 20 orders, 383 IVs in 35 families, and 1,372 IVs in 131 genera. In addition, all IVs were more strongly associated with exposure than with outcome (*p*_exposure_ < *p*_outcome_), and all F-statistics were greater than 10. Details of the IVs are presented in Supplementary Table S1.

### 3.2. MR analysis

**Figure 2:**
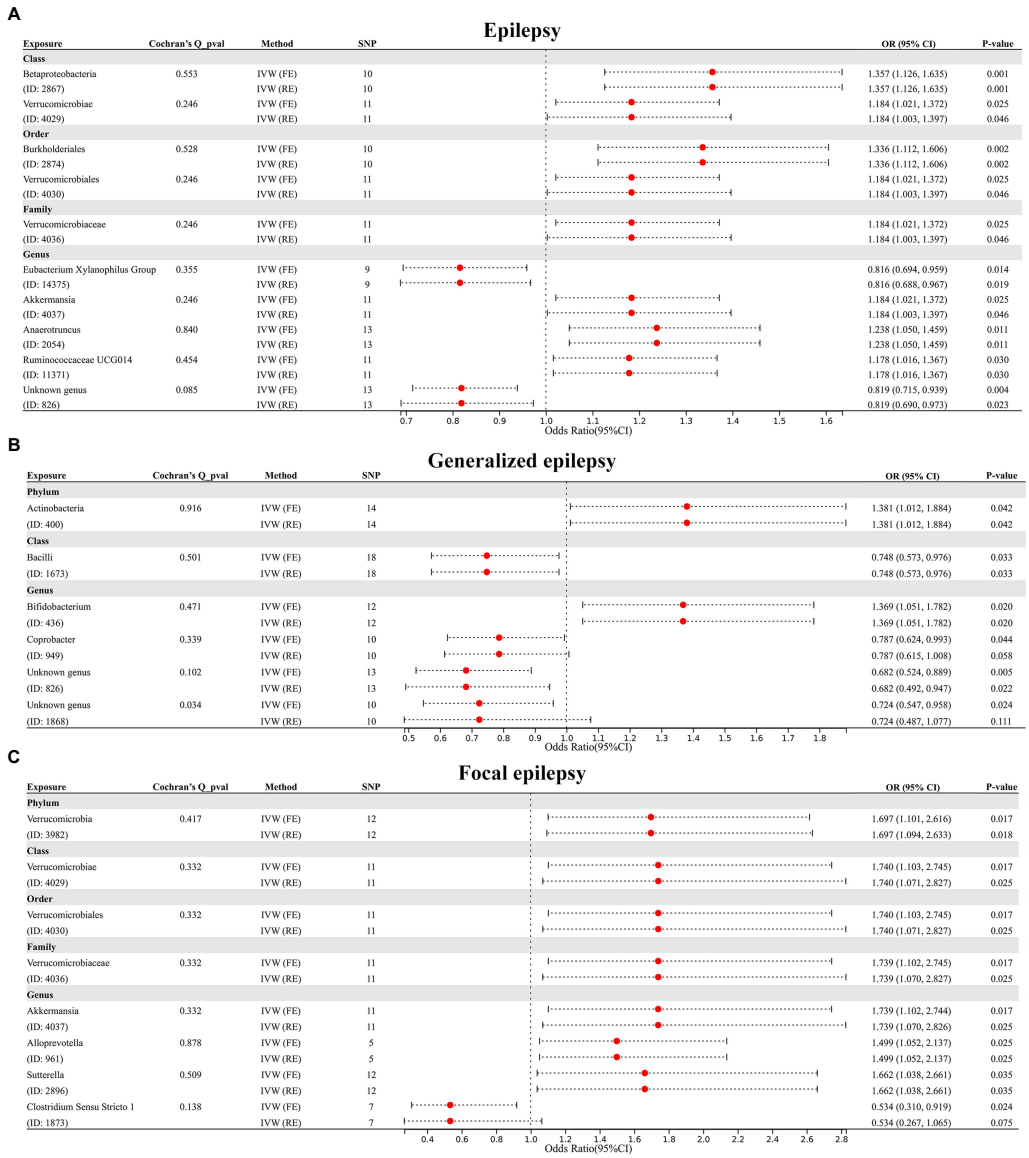
**(A)** Forest plot of GM taxa associated with epilepsy identified by IVW_FE method. **(B)** Forest plot of GM taxa associated with generalized epilepsy identified by IVW_FE method. **(C)** Forest plot of GM taxa associated with focal epilepsy identified by IVW_FE method.

First, we performed MR analysis to assess the causal association of 211 GM taxa at five levels with epilepsy. The results assessed by the IVW-FE showed that class *Betaproteobacteria* (ID: 2867), class *Verrucomicrobiae* (ID: 4029), order *Burkholderiales* (ID: 2874), order *Verrucomicrobiales* (ID: 4030), family *Verrucomicrobiaceae* (ID: 4036), genus *Akkermansia* (ID: 4037), genus *Anaerotruncus* (ID: 2054) and genus *Ruminococcaceae UCG 014* (ID: 11371) were associated with an increased risk for epilepsy, while genus *Eubacterium Xylanophilus Group* (ID: 14375) and genus *Unknown genus* (ID: 826) were associated with a decreased risk for epilepsy (Figure 2A). Furthermore, the results of Cochran’s Q test indicated the absence of heterogeneity. After applying the Bonferroni correction, class *Betaproteobacteria* (ID: 2867) [OR = 1.357 (1.126, 1.635), *p* = 0.001] and order *Burkholderiales* (ID: 2874) [OR = 1.336 (1.112, 1.606), *p* = 0.002] remained risk factors for epilepsy.

Subsequently, we further evaluated the causal association of 211 GM taxa with generalized epilepsy using the IVW-FE method. The results showed that phylum *Actinobacteria* (ID: 400) and genus *Bifidobacterium* (ID: 436) were associated with an increased risk for generalized epilepsy, while class *Bacilli* (ID: 1673), genus *Coprobacter* (ID: 949), genus *Unknown genus* (ID: 826) and genus *Unknown genus* (ID: 1868) were associated with a decreased risk for generalized epilepsy (Figure 2B). However, after Bonferroni correction, the causal effect of these GM taxa on generalized epilepsy was insignificant. Furthermore, the results of Cochran’s *Q*-test suggested heterogeneity in the MR analysis of Genus *Unknown genus* (ID: 1868); thus, the IVW random effect (RE) was applied to explain the causal association of this GM taxon with generalized epilepsy, with results indicating no causal association.

Finally, we assessed the causal association of 211 GM taxa with focal epilepsy using the IVW-FE method. The results showed that phylum *Verrucomicrobia* (ID: 3982), class *Verrucomicrobiae* (ID: 4029), order *Verrucomicrobiales* (ID: 4030), family *Verrucomicrobiaceae* (ID: 4036), genus *Akkermansia* (ID: 4037), genus *Alloprevotella* (ID: 961), and genus *Sutterella* (ID: 2896) were associated with an increased risk for focal epilepsy, while genus *Clostridium Sensu Stricto 1* (ID: 1873) was associated with a decreased risk for focal epilepsy (Figure 2C). The results of Cochran’s Q test suggested no heterogeneity in the MR analysis. However, after Bonferroni correction, the causal effect of these GM taxa on generalized epilepsy was insignificant.

**Figure 3:**
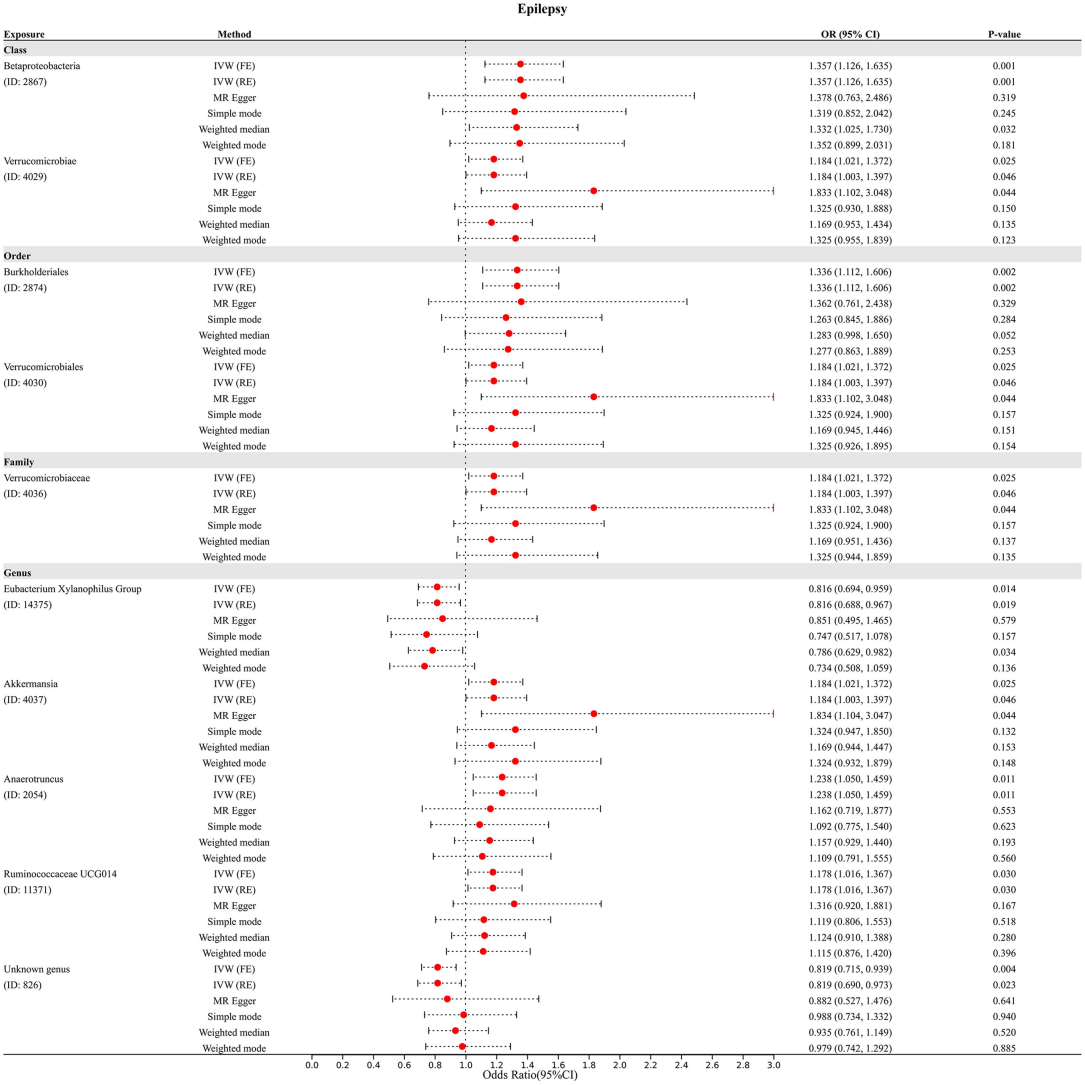
Diverse Mendelian randomization (MR) results for 10 GM taxa causally associated with epilepsy.

**Figure 4:**
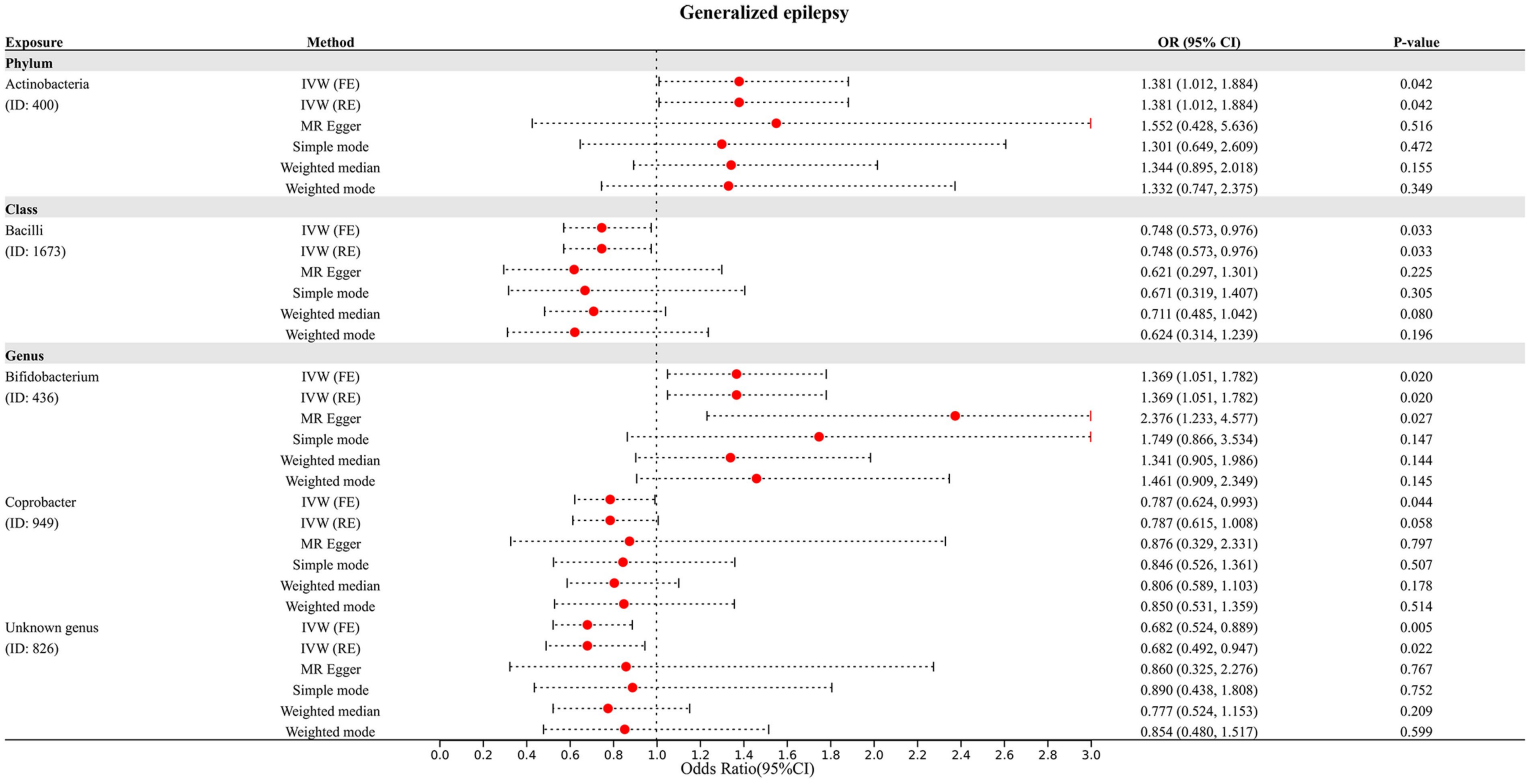
Diverse MR results for 5 GM taxa causally associated with generalized epilepsy.

**Figure 5:**
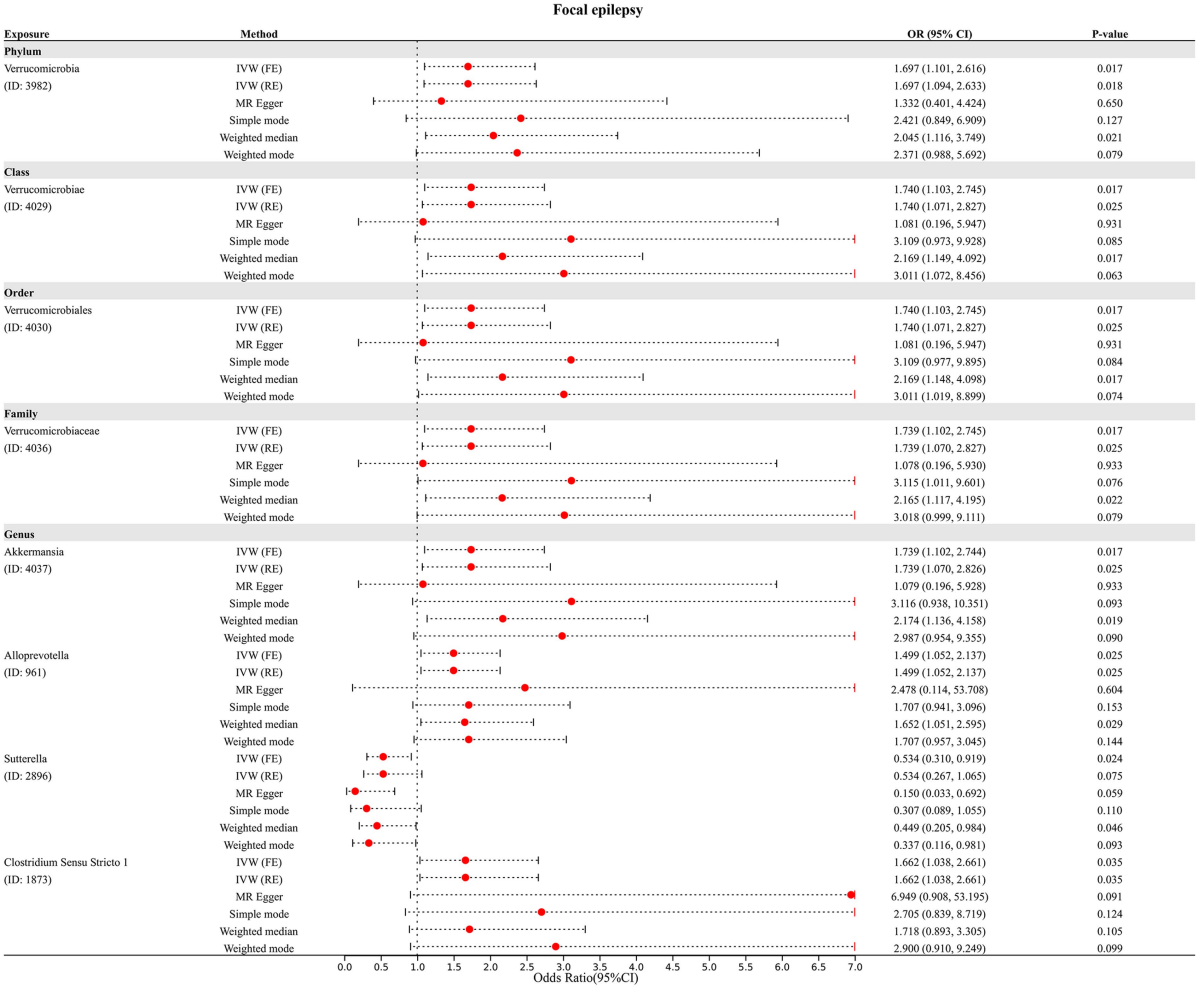
Diverse MR results for 8 GM taxa causally associated with focal epilepsy.

**Figure 6:**
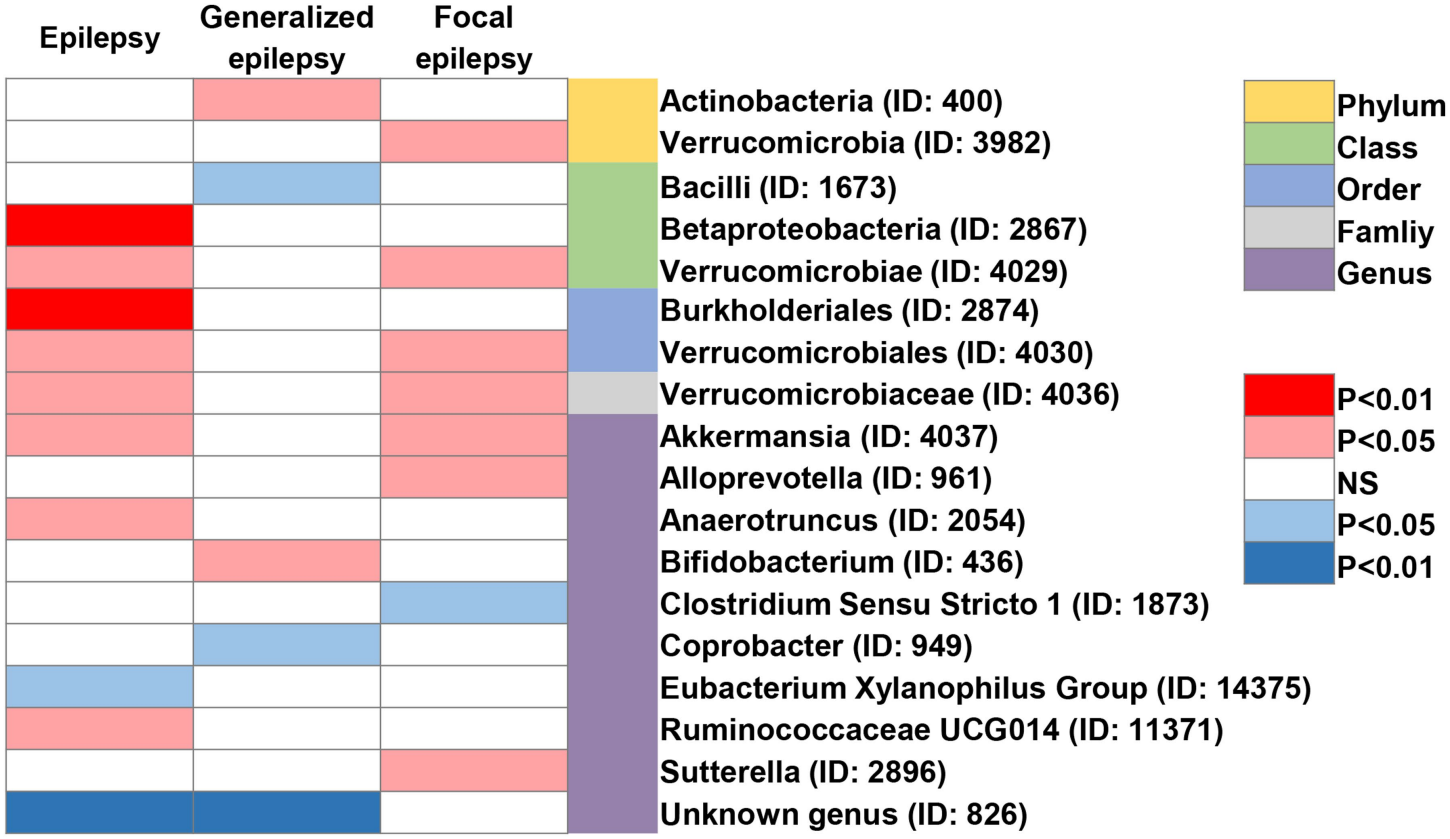
Heat map of GM taxa causally associated with epilepsy, generalized epilepsy, and focal epilepsy identified by IVW method. Red represents risk factors, while blue represents protective factors.

In addition, four additional methods, MR-Egger, weighted median, simple mode, and weighted mode, were performed to assess the causal effect of these GM taxa on epilepsy (Figure 3), generalized epilepsy (Figure 4), and focal epilepsy (Figure 5). Similarly, the results were parallel to the IVW results (Supplementary Figure S1). The heat map visualized the causal association of GM taxa identified in our study with epilepsy, generalized epilepsy, and focal epilepsy (Figure 6).

### 3.3. Sensitivity analysis

The results of the MR-Egger intercept test and MR-PRESSO global test showed that there was no horizontal pleiotropy (*p*_MR-Egger intercept_ > 0.05 and global *p*_MR-PRESSO_ > 0.05) in (i) IVs of 10 GM taxa associated with epilepsy (Table 2), (ii) IVs of 5 GM taxa associated with generalized epilepsy (Table 3), and (iii) IVs of 8 GM taxa associated with focal epilepsy (Table 4). In addition, the leave-one-out analysis indicated the robustness of the MR results since excluding any one IV did not shift the overall results (Supplementary Figure S2).

**Table 2:** Horizontal pleiotropy analysis for IVs of 10 GM taxa associated with epilepsy.

Exposure	MR-Egger intercept test			MR-PRESSO global test	
	Egger_intercept	SE	*p*-value	RSS obs	*p*-value
**Class**					
Betaproteobacteria (ID: 2867)	−0.001	0.021	0.959	9.543	0.578
Verrucomicrobiae (ID: 4029)	−0.036	0.020	0.111	15.497	0.266
**Order**					
Burkholderiales (ID: 2874)	−0.001	0.021	0.948	9.840	0.551
Verrucomicrobiales (ID: 4030)	−0.036	0.020	0.111	15.497	0.271
**Family**					
Verrucomicrobiaceae (ID: 4036)	−0.036	0.020	0.112	15.499	0.274
**Genus**					
Eubacterium Xylanophilus Group(ID: 14375)	−0.004	0.023	0.876	11.147	0.433
Akkermansia (ID: 4037)	−0.036	0.020	0.111	15.484	0.280
Anaerotruncus (ID: 2054)	0.005	0.017	0.788	8.363	0.865
Ruminococcaceae UCG014(ID: 11371)	−0.010	0.016	0.522	11.928	0.479
Unknown genus (ID: 826)	−0.007	0.024	0.771	23.163	0.105

SE, standard error; RSS, residual sum of squares.

**Table 3:** Horizontal pleiotropy analysis for IVs of 5 GM taxa associated with generalized epilepsy.

Exposure	MR-Egger intercept test			MR-PRESSO global test	
	Egger_intercept	SE	*p*-value	RSS obs	*p*-value
**Phylum**					
Actinobacteria (ID: 400)	−0.007	0.040	0.858	7.596	0.920
**Class**					
Bacilli (ID: 1673)	0.015	0.028	0.605	18.510	0.532
**Genus**					
Bifidobacterium (ID: 436)	−0.049	0.027	0.102	12.796	0.483
Coprobacter (ID: 949)	−0.012	0.053	0.830	12.348	0.384
Unknown genus (ID: 826)	−0.023	0.046	0.630	21.511	0.128

SE, standard error; RSS, residual sum of squares.

**Table 4:** Horizontal pleiotropy analysis for IVs of 8 GM taxa associated with focal epilepsy.

Exposure	MR-Egger intercept test			MR-PRESSO global test	
	Egger_intercept	SE	*p*-value	RSS obs	*p*-value
**Phylum**					
Verrucomicrobia (ID: 3982)	0.023	0.054	0.678	13.273	0.450
**Class**					
Verrucomicrobiae (ID: 4029)	0.039	0.068	0.581	13.605	0.379
**Order**					
Verrucomicrobiales (ID: 4030)	0.039	0.068	0.581	13.605	0.364
**Family**					
Verrucomicrobiaceae(ID: 4036)	0.039	0.068	0.579	13.610	0.341
**Genus**					
Akkermansia (ID: 4037)	0.039	0.068	0.579	13.608	0.364
Alloprevotella (ID: 961)	−0.070	0.217	0.768	1.914	0.895
Clostridium *Sensu Stricto* 1(ID: 1873)	0.137	0.078	0.138	12.978	0.187
Sutterella (ID: 2896)	−0.098	0.069	0.187	12.428	0.525

SE, standard error; RSS, residual sum of squares.

### 3.4. Replicated analysis after removing confounders-related IVs

Among the IVs of 10 GM taxa associated with epilepsy, rs4936098 was associated with obesity and rs2321387 with education level. In addition, among the IVs of 5 GM taxa associated with generalized epilepsy, rs12634544, rs182549, rs1397793, rs7570971, rs35344081, and rs35344081 were associated with obesity; rs182549 with diabetes mellitus; and rs2952251 with smoking. Furthermore, among the IVs of 8 GM taxa associated with focal epilepsy, rs4936098 was associated with obesity, and rs2321387 with education level. After removing these SNPs from the IVs, the causal associations of these GM taxa were re-evaluated by the IVW-FE method. The results showed that, except for phylum *Actinobacteria* (ID: 400), the causal effects of the above GM taxa remained significant (Table 5).

**Table 5:** Replicated MR analysis by IVW method after removing confounders-related IVs.

Exposure	Outcome	*p*-value	OR (95% CI)
**Phylum**			
Actinobacteria (ID: 400)	Generalized epilepsy	0.132	1.318 (0.920, 1.889)
Verrucomicrobia (ID: 3982)	Focal epilepsy	0.017	1.697 (1.101, 2.616)
**Class**			
Betaproteobacteria(ID: 2867)	Epilepsy	0.001	1.381 (1.136, 1.679)
Verrucomicrobiae (ID: 4029)	Epilepsy	0.030	1.186 (1.016, 1.384)
Bacilli (ID: 1673)	Generalized epilepsy	0.025	0.718 (0.537, 0.960)
Verrucomicrobiae (ID: 4029)	Focal epilepsy	0.008	1.900 (1.178, 3.063)
**Order**			
Burkholderiales (ID: 2874)	Epilepsy	0.002	1.359 (1.121, 1.648)
Verrucomicrobiales (ID: 4030)	Epilepsy	0.030	1.186 (1.016, 1.384)
Verrucomicrobiales (ID: 4030)	Focal epilepsy	0.008	1.900 (1.178, 3.063)
**Family**			
Verrucomicrobiaceae (ID: 4036)	Epilepsy	0.030	1.186 (1.016, 1.385)
Verrucomicrobiaceae (ID: 4036)	Focal epilepsy	0.008	1.900 (1.178, 3.063)
**Genus**			
Eubacterium Xylanophilus Group (ID: 14375)	Epilepsy	0.014	0.816 (0.694, 0.959)
Akkermansia (ID: 4037)	Epilepsy	0.030	1.186 (1.017, 1.385)
Anaerotruncus (ID: 2054)	Epilepsy	0.011	1.238 (1.050, 1.459)
Ruminococcaceae UCG014(ID: 11371)	Epilepsy	0.030	1.178 (1.016, 1.367)
Unknown genus (ID: 826)	Epilepsy	0.004	0.819 (0.715, 0.939)
Bifidobacterium (ID: 436)	Generalized epilepsy	0.036	1.389 (1.021, 1.890)
Coprobacter (ID: 949)	Generalized epilepsy	0.044	0.787 (0.624, 0.993)
Unknown genus (ID: 826)	Generalized epilepsy	0.005	0.682 (0.524, 0.889)
Akkermansia (ID: 4037)	Focal epilepsy	0.008	1.899 (1.178, 3.062)
Alloprevotella (ID: 961)	Focal epilepsy	0.025	1.499 (1.052, 2.137)
Clostridium *Sensu Stricto* 1(ID: 1873)	Focal epilepsy	0.024	0.534 (0.310, 0.919)
Sutterella (ID: 2896)	Focal epilepsy	0.032	1.715 (1.048, 2.806)

OR, odds ratio; CI, confidence interval.

## 4. Discussion

Our study comprehensively assessed the causal effect of 211 GM taxa (from phylum to genus level) on epilepsy and its sub-types. Finally, we identified a total of 23 causal relationships, of which 21 were nominal causal relationships, and two were strong causal relationships, thus highlighting the importance of GMs in epilepsy.

Accumulating evidence has suggested crosstalk between GMs and the central nervous system (CNS) (Cryan and Dinan, 2012). Investigations have shown that GMs play a vital role in the development of the enteric nervous system, blood–brain barrier, and glial cells, which are all important for cognitive development and behavior regulation (Braniste et al., 2014; Collins et al., 2014). Various neurological disorders, including multiple sclerosis (Jangi et al., 2016), autism (Mulle et al., 2013), Alzheimer’s disease (Jiang et al., 2017), and Parkinson’s disease (Parashar and Udayabanu, 2017), have been linked to intestinal dysbiosis. Recent findings also suggest that GMs may also play a role in epilepsy (Russo, 2022). Several studies have examined the effect of the KD, a treatment for refractory epilepsy, on GMs to explore the potential mechanisms of GMs in KD treatment (Lum et al., 2020). However, it is inconclusive which GM taxa have the most significant impact on epilepsy. As one-third of patients with epilepsy are diagnosed with refractory epilepsy (Dahlin and Prast-Nielsen, 2019), exploring biomarkers of epilepsy on the GMs level could offer promising alternative treatment options and potentially prevent the need for invasive treatments such as vagus nerve stimulation (VNS) or epilepsy surgery (Braakman and van Ingen, 2018).

Our study identified two strong causal relationships. *Class Betaproteobacteria* (OR = 1.357, 95% CI: 1.126–1.635, *p* = 0.001) and *Order Burkholderiales* (OR = 1.336, 95% CI: 1.112–1.606, *p* = 0.002) significantly elevated the epilepsy risk after Bonferroni correction. *Burkholderiales*, an order of *Betaproteobacteria*, was found to have a potential impact on epilepsy from our MR study, which was consistent with the findings of some previous investigations. For instance, Safak et al. identified the *genus Delftia* and *genus Lautropia*, which are members of *Burkholderiales*, to be significantly higher in the intestine of epilepsy patients versus healthy individuals (Safak et al., 2020). In addition, another genus of *Burkholderiales*, *Sutterella*, which was reported with increased intestinal abundance in adult patients with epilepsy (Dong et al., 2022), was also identified in our study to be nominally associated with an increased risk of focal epilepsy. The present MR study could provide evidence and confidence for the increased level of genera belonging to *Order Burkholderiales* in the intestines of epilepsy patients.

It’s important to note that the Bonferroni correction can result in false negatives. Our findings showed 21 GM taxa with nominal causal connections, but these correlations vanished after applying the Bonferroni correction. This may be due to the crosstalk between the gut-brain axis being usually coordinated by multiple factors and that the role of a single microbiota in the genus level in causing disease may not be as important as previously thought. In fact, several GM taxa with nominal causal relationships identified in this study corroborate the findings of previous research. For instance, Huang et al. revealed that patients with cerebral palsy and epilepsy contained a higher proportion, in comparison to healthy controls, of *Bifidobacterium* and *Akkermansia* (Huang et al., 2019). In addition, Gong and colleagues identified *Bifidobacterium*, *Ruminococcaceae UCG 014*, and *Akkermansia* at the genus level were increased in patients with epilepsy compared to healthy controls (Gong et al., 2020). Further, Lee et al. identified *Enterococcus faecium* (species of class *Bacilli*), *Bifidobacterium longum* (species of genus *Bifidobacterium*), and *Eggerthella lenta* (species of phylum *Actinobacteria*) as biomarkers for drug-resistant epilepsy (Lee et al., 2021). Although only nominal causal associations were identified at the genus level for these GM taxa, the coordination and crosstalk between various GM taxa remain worthy of in-depth study in the future.

The mechanisms involved in the relationship between GMs and epilepsy have not been fully determined. However, some evidence suggests potential mechanisms. (i) Studies have reported that GMs can alter neurotransmitter levels such as glutamate, gamma-aminobutyric acid (GABA), 5-hydroxytryptamine (5-HT) (Mittal et al., 2017), as well as increase levels of cytokines, chemokines, such as TNF⍺ and MCP-1, lipopolysaccharides (LPS) which led to generalized immune activation or inflammation (Blander et al., 2017), contributing to the risk of seizures. (ii) GMs have been demonstrated to interact with gut-derived metabolites, resulting in both beneficial and detrimental mechanisms for the central nervous system (Tran and Mohajeri, 2021). (iii) In addition, GMs can affect the hypothalamic–pituitary–adrenal (HPA) axis (Sudo et al., 2004) and the levels of brain-derived neurotrophic factor (BDNF) (Maqsood and Stone, 2016), which promote seizure propensity. (iv) GMs also regulate peripheral metabolites and central neurotransmitter metabolism, which affect seizure susceptibility (Lum et al., 2020). Nevertheless, the specific mechanism and crosstalk between different GM taxa remain to be verified by future studies.

The limitations of the present study should be noted: (i) Since the number of IVs fulfilling the strict threshold (*p* < 5 × 10^−8^) was extremely small, a relatively lenient threshold (*p* < 1 × 10^−5^) was adopted for screening IVs. (ii) This study included individuals of essentially European ancestry, so extrapolating the findings to other populations is limiting. (iii) The number of cases of the two subtypes of epilepsy under strict definition (generalized epilepsy and focal epilepsy) is relatively small, so future analysis based on a larger sample size of GWAS summary data is necessary to increase the confidence of the results. (iv) The GM-related GWAS summary-level dataset included in this study was based on 16S rRNA sequencing, and thus further analysis based on large-scale studies with more advanced methods, such as metagenomics sequencing, is required in the future in order to evaluate the species-level. (v) Current studies of GMs have focused only on bacteria; however, other types of GMs may also have potential functions.

## 5. Conclusion

Overall, by performing MR analysis of the causal effects of 211 GM taxa on epilepsy and its sub-types, we finally identified 21 nominal causal relationships and two strong causal relationships. Among them, *Class Betaproteobacteria* and *Order Burkholderiales* are significantly associated with increased epilepsy risk. However, it is essential to recognize that since the present study was conducted based on the GWAS summary-level dataset generated from 16S rRNA sequencing, further in-depth analyses based on more advanced large-scale studies generated from metagenomics sequencing are necessary. Nevertheless, our findings may provide helpful biomarkers for disease progression and potential candidate therapeutic targets for epilepsy.

## Data availability statement

Publicly available datasets were analyzed in this study. This data can be found at: https://mibiogen.gcc.rug.nl/, https://r7.finngen.fi/.

## Ethics statement

Publicly available de-identified data from participant studies approved by an ethical standards committee were used in this study. Therefore, no additional separate ethical approval was required for this study.

## Author contributions

YZ designed the study, analyzed the data, and wrote the manuscript. SC assisted in analyzing the data and revising the manuscript. HY critically read and edited the manuscript. All authors contributed to the article and approved the submitted version.

## Funding

This research was funded by the Natural Science Foundation of Hunan Province (2022JJ70069).

## Conflict of interest

The authors declare that the research was conducted in the absence of any commercial or financial relationships that could be construed as a potential conflict of interest.

## Publisher’s note

All claims expressed in this article are solely those of the authors and do not necessarily represent those of their affiliated organizations, or those of the publisher, the editors and the reviewers. Any product that may be evaluated in this article, or claim that may be made by its manufacturer, is not guaranteed or endorsed by the publisher.

## Footnotes

^1^https://mibiogen.gcc.rug.nl/

^2^https://r7.finngen.fi/

^3^http://www.phenoscanner.medschl.cam.ac.uk/

^4^https://mrcieu.github.io/TwoSampleMR/

^5^https://github.com/rondolab/MR-PRESSO
