# Supplementary material for: Roles of gut microbiome in epilepsy risk: A Mendelian randomization study
Source: Front Microbiol. 2023 Feb 27;14:1115014. doi: 10.3389/fmicb.2023.1115014 (PMC10010438; doi:10.3389/fmicb.2023.1115014)
Supplement: Supplementary file 1 [file Data_Sheet_1.PDF]

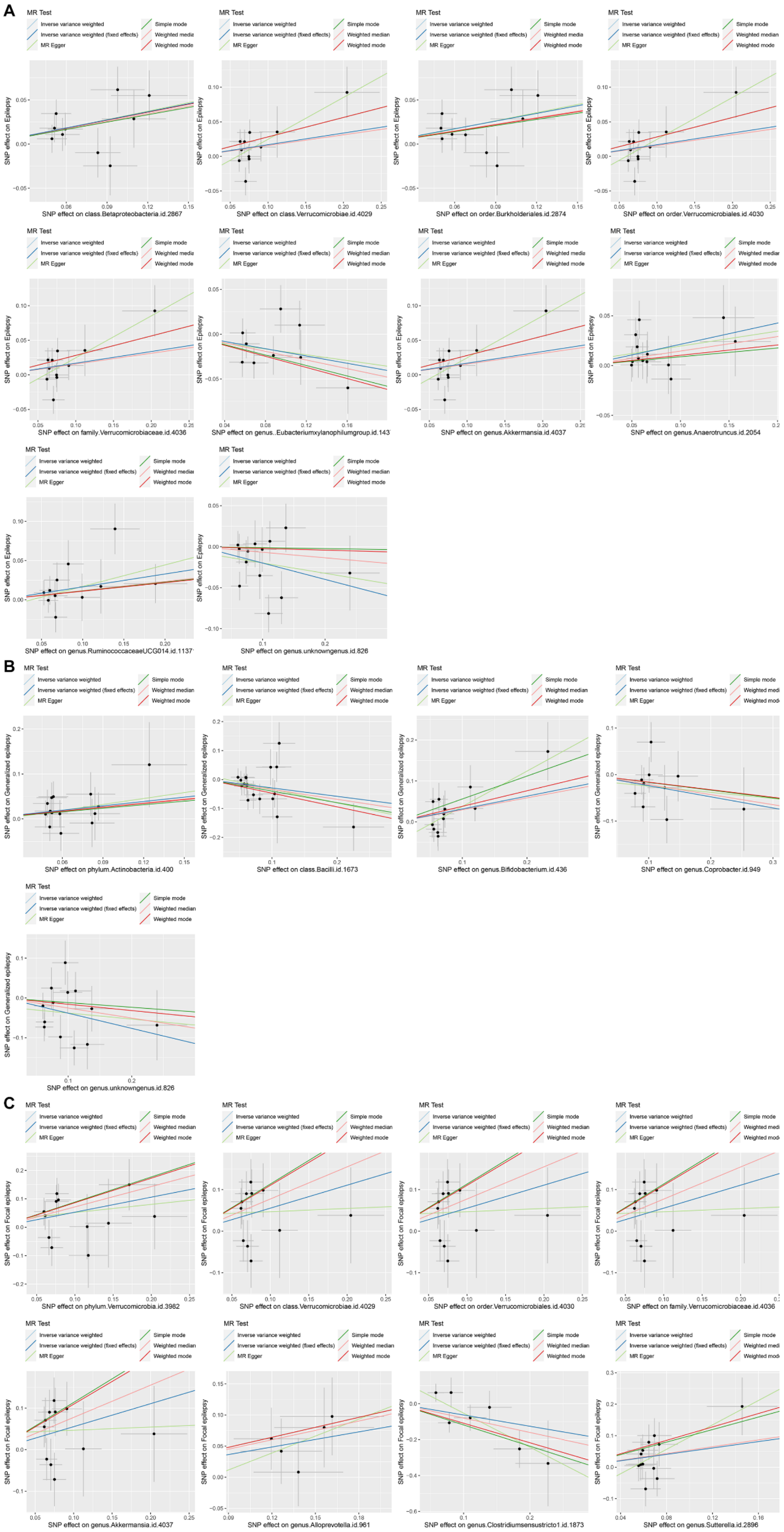

Supplementary Figure S1. Scatter plot of MR results. (A) scatter plot of genetic correlations of 10 GM taxa and epilepsy using different MR methods. (B) scatter plot of genetic correlations of 5 GM taxa and generalized epilepsy using different MR methods. (C) scatter plot of genetic correlations of 8 GM taxa and focal epilepsy using different MR methods.
