# Supplementary material for: Roles of gut microbiome in epilepsy risk: A Mendelian randomization study
Source: Front Microbiol. 2023 Feb 27;14:1115014. doi: 10.3389/fmicb.2023.1115014 (PMC10010438; doi:10.3389/fmicb.2023.1115014)
Supplement: Supplementary file 2 [file Data_Sheet_2.PDF]

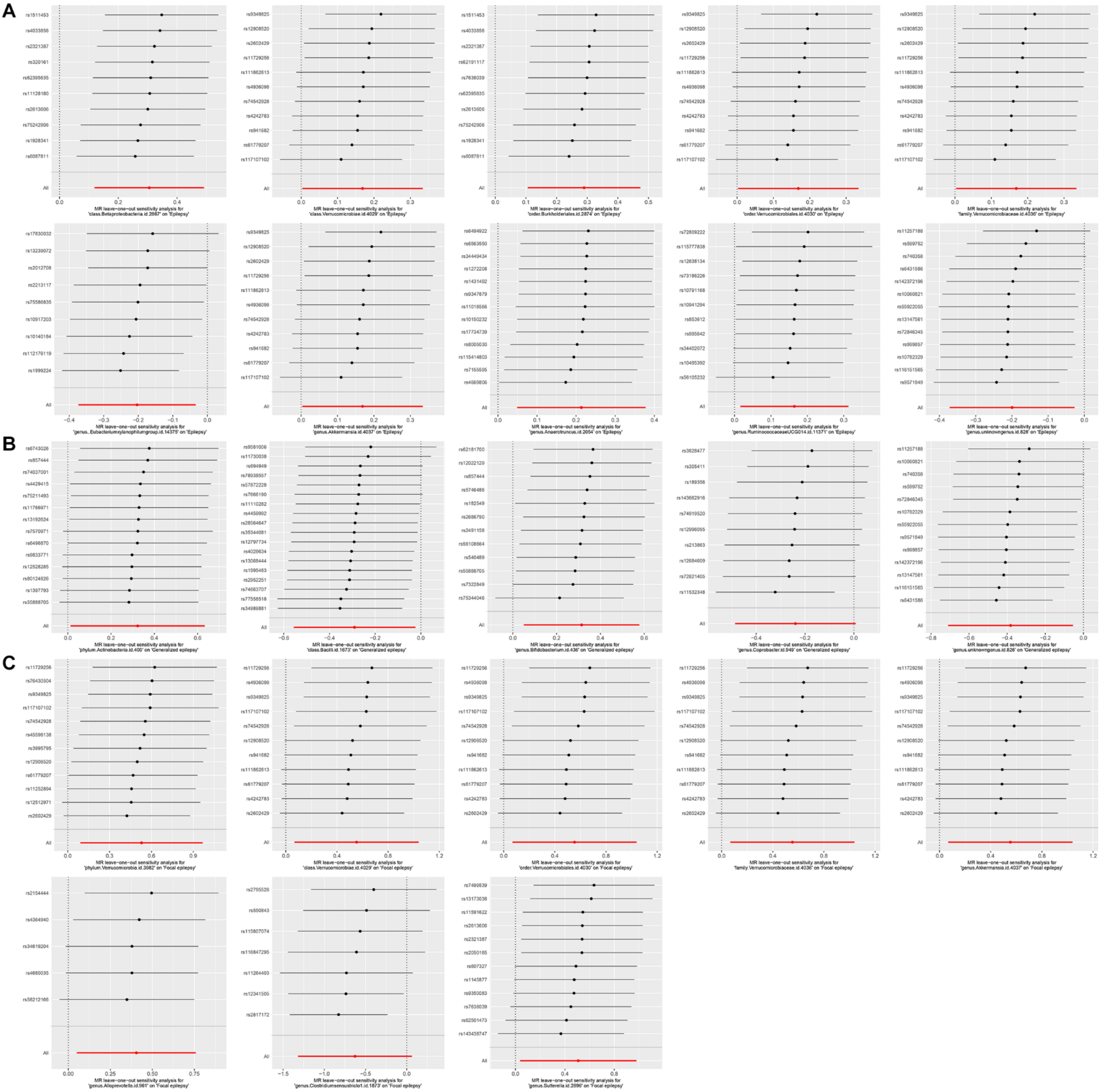

Supplementary Figure S2. Leave-one-out analysis for (A) 10 GM taxa on epilepsy, (B) 5 GM taxa on generalized epilepsy, and (C) 8 GM taxa on focal epilepsy.
